# Exogenous putrescine application imparts salt stress-induced oxidative stress tolerance via regulating antioxidant activity, potassium uptake, and abscisic acid to gibberellin ratio in *Zinnia* flowers

**DOI:** 10.1186/s12870-024-05560-0

**Published:** 2024-09-16

**Authors:** Meisam Mohammadi, Delaram Nezamdoost, Fatemeh Khosravi Far, Faisal Zulfiqar, Ghasem Eghlima, Fatame Aghamir

**Affiliations:** 1 Department of Horticulture, Faculty of Agriculture, Ilam University, Ilam, Iran; 2 Department of Horticulture, Faculty of Agriculture, University of Zanjan, Zanjan, Iran; 3 Department of Horticultural Sciences, Faculty of Agriculture and Environment, The Islamia University of Bahawalpur, Bahawalpur, 63100 Pakistan; 4 Department of Agriculture, Medicinal Plants and Drugs Research Institute, Shahid Beheshti University, Tehran, Iran

**Keywords:** Plant height, Antioxidant enzymes, Proline, Fresh weight, Salt stress

## Abstract

This research was conducted to investigate the efficacy of putrescine (PUT) treatment (0, 1, 2, and 4 mM) on improving morphophysiological and biochemical characteristics of *Zinnia elegans* “State Fair” flowers under salt stress (0, 50, and 100 mM NaCl). The experiment was designed in a factorial setting under completely randomized design with 4 replications. The results showed that by increasing the salt stress intensity, the stress index (SSI) increased while morphological traits such as plant height decreased. PUT treatments effectively recovered the decrease in plant height and flower quality compared to the not-treated plants. Treatment by PUT 2 mM under 50 and 100 mM salt stress levels reduced the SSI by 28 and 35%, respectively, and increased plant height by 20 and 27% compared to untreated plants (PUT 0 mM). 2 mM PUT treatment also had the greatest effect on increasing fresh and dry biomass, number and surface area of leaves, flower diameter, internodal length, leaf relative water content, protein contents, total chlorophyll contents, carotenoids, leaf potassium (K^+^) content, and K^+^/Na^+^ ratio in treated plants compared to untreated control plants. The treatment of 2 mM PUT decreased the electrolyte leakage, leaf sodium (Na^+^) content, H_2_O_2_, malondialdehyde, and proline content. Furthermore, PUT treatments increased the activity of defense-related enzymes including catalase (CAT), peroxidase (POD), superoxide dismutase (SOD), and phenylalanine ammonium lyase (PAL), and reduced the abscisic acid (ABA) content while increased the level of gibberellin (GA) content compared to untreated samples under all different levels of salinity stress. In this research, enhancing the plant’s antioxidant system, increasing K^+^ absorption, K^+^/Na^+^ ratio, and reducing the ABA/GA ratio are likely the most important mechanisms of PUT treatment, which improved growth, and maintained the visual quality of zinnia flowers under salt stress conditions.

## Introduction

Zinnia (*Zinnia elegans*) is a valuable cut flower crop of the *Asteraceae* family which is widely grown as a popular warm-season annual plant worldwide [1]. Zinnia can be used as a cut flower, garden plant, or potted plant. It has a wide variety of flower colors, and shapes, which makes it a popular choice for use in landscaping, gardening, and as a cut flower crop [2]. Zinnia is a low-maintenance plant and can be grown in a wide range of soil and environmental conditions. Its low-maintenance and high ornamental value has made it a widely used ornamental plant in landscape settings [3]. Salt stress tolerance in zinnia varies among cultivars. Research examining the salt tolerance of zinnia cultivars has shown that, overall, this plant is sensitive to salt stress, and its cultivation in areas with saline soil or water sources leads to a significant decrease in its production [1].

Salt stress in a soil is a condition when the electrical conductivity (EC) of a saturated soil extract is 4 dS/m or higher. It is an environmental stress factor that reduces plant’s growth and their final performance by affecting various physiological and biochemical processes [4]. Salt stress mainly occurs in dry, semi-dry, and coastal regions where rainfall is low, and evaporation and transpiration rates are at high levels [5]. Nevertheless, the persistence of salt stress in soil or water sources results from natural weathering of saline rocks, human activities such as improper irrigation methods with saline water, and constantly changing weather conditions. Today, approximately 10% of the total land area and 50% of the freshwater sources worldwide are affected by salt stress. Soil or water salt stress also poses an increasing threat to the growth and performance of ornamental plants used in gardening and landscaping or produced as cut flowers [5, 6]. Salt stress affects plants in various ways, influencing water uptake, antioxidant content, chlorophyll levels, and subsequently photosynthesis. High salt levels in the soil cause osmotic stress and make water absorption more difficult for plants by clogging the root pores. Additionally, the accumulation of salt ions in the plant tissues causes oxidative stress that leads to the uncontrolled production of reactive oxygen species (ROS). Under such a situation, plants by default increase the activity of defense-related antioxidants to a certain level depending on the capacity of the plant [7].

The vigor and visual quality of the ornamental plants are considered as vital aesthetic indicators in the ornamental industry. The appearance of stress symptoms such as yellowing of leaves, wilting, etc., reduces both the performance and beauty of ornamental plants, and hence efforts must be made to preserve the ornamental plants in the face of such environmental stresses under the threat of climate change [1]. Plant scientists have discovered various solutions to manage salt stress in plants. These solutions include the use of chemical substances chemical compounds, soil and water amendment with organic materials, optimal irrigation technologies, planting salt-resistant species, and using plant growth regulators (PGRs), signaling molecules, osmoprotectants and biostimulants to regulate plant growth and development. These methods help plants to perform better under salt stress conditions [1, 8].

Polyamines are considered as one of the useful and environmentally compatible compounds for reducing the effects of environmental-related stresses on crop plants. Among the most important polyamines are putrescine (PUT), spermidine, and spermine. Polyamines are organic compounds with multiple amine groups and very low molecular weights that play essential roles in physiological processes, cell growth, and stress responses. PUT (C_4_H_12_N_2_) helps by maintaining ionic homeostasis to limit the uptake of toxic sodium ions and increase the absorption of vital potassium ions to balance the ionic stress [9]. Additionally, PUT helps regulate osmotic balance, which is crucial for combating osmotic stress resulting from high soil salt stress. It also enhances the plant’s antioxidant defense system and effectively eliminates harmful ROS produced during salt stress, thereby minimizing oxidative damage to plant cells [10]. PUT also plays a role in stomatal regulation and helps control water loss from plant leaves under salt-stress conditions. This dual role of PUT helps plants adapt to salt stress conditions [11].

It has been reported that PUT, as a polyamine, helps plants cope with salt stress by regulating osmotic balance, acting as an antioxidant, maintaining ion homeostasis, promoting root growth and cell division, and enhancing plant adaptability to high salt stress conditions [12]. In a study, it was reported that PUT regulates stomatal conductance in cucumber under salt stress conditions, as evidenced by PUT foliar spraying inducing H_2_O_2_ signaling and inhibiting ABA accumulation in leaves, resulting in increased stomatal conductivity in cucumber leaves under salt stress, ultimately improving plant performance under stress conditions [13]. In another study, the use of PUT increased nitric oxide synthase production and subsequently enhanced cold stress tolerance in Anthurium flowers [14]. In this regard, a study using melatonin in the form of seed priming in Zinnia flowers has been reported to significantly improve photosynthesis efficiency and plant performance by minimizing cellular damage from salt stress and scavenging of ROS [1]. Additionally, in another study, the impact of spermine, gamma-aminobutyric acid (GABA), and beta-aminobutyric acid treatments at pre- and post-harvest on the vase life and quality of Gerbera cut flowers was investigated. The results showed that spermine and GABA treatments, especially at pre-harvest, increased the vase life and improved the postharvest quality of the cut flowers. These treatments not only had positive effects on the quality characteristics of the flowers but also, by enhancing the antioxidant system and increasing enzymatic activity, reduced the activity of toxic ROS and senescence process during the flowers post-harvest conditions, ultimately resulting in reduced wilting and bending of the cut flowers during postharvest [15].

It was hypothesized that the use of PUT can ameliorate the ill impacts of salt stress on ornamental zinnia plants. Therefore, considering the positive effects of polyamines on physiological processes related to plant response to environmental stresses in previous reports, the present study was designed to investigate the impact of PUT foliar spraying on morphophysiological and biochemical characteristics of “State Fair” Zinnia ornamental plants under salt stress conditions.

## Materials and methods

### Plant materials and treatments

This study was conducted in a research greenhouse at Ilam University, Ilam, Iran (33° 39ʹ 23ʺ N, 46° 22ʹ 37ʺ E, H:1458 m), from November 2022 to January 2023. 5 seeds of Zinnia ‘State Fair’ cultivar (produced by Pakan Seed Company, Isfahan, Iran. https://www.pakanbazr.com/) were sown in plastic pots of 4 L capacity. At the stage of appearance of cotyledon leaves, 2 seedlings were kept in each pot and the rest of the seedlings were removed. The growing media consisted of a uniform mixture of 50% agricultural soil (silt 48%, clay 40%, sand 12%), 25% decomposed peat (Holland Free Peat), and 25% perlite. The plants were irrigated with normal water until they reached the four-true-leaf stage.

At the four-true-leaf stage, the plants were completely foliar sprayed twice with concentrations of 0, 1, 2, and 4 mM of PUT (Putrescine dihydrochloride, Sigma-Aldrich, USA), with a 4-day interval between applications. To enhance treatment absorption, Tween 10 at a 0.5% ratio was used as a surfactant. The treatments were applied by manual spraying of the whole surface of the plants, and distilled water was used to treat control plants (PUT 0 mM). Then, 7 days after the application of PUT treatments, salt stress at concentrations of 0, 50, and 100 mM, along with 250 mL irrigation water, was applied. Sodium chloride (NaCl, Sigma-Aldrich, USA) was used for the salt treatments. After each saline irrigation, regular water irrigation was used once to prevent salt accumulation. The greenhouse temperature during the growth period was 30–32 °C during the day and 18–20 °C at night. Initially, the photoperiod was 10 h of light and 14 h of darkness, but during the flowering period, with the change of season, the photoperiod was approximately equal (12/12 h). Plant sampling was performed after entering the flowering phase and flower emergence (68 days after sowing the seeds). The studied traits included some momMrphological, physiological, and biochemical characteristics.

### Measurement of traits

#### Morphological characteristics

At the full flowering stage (68 days after sowing the seeds), plant height and internode length were measured using a ruler (mm). Flower diameter was measured by a digital caliper. The number of leaves per plant was counted. Leaf surface area (mm^2^) was measured using a digital leaf area meter (Abi Aca Co, AM-350). Plant fresh and dry weights were measured using a digital scale (Smart Weigh, SWS-100). For dry weight measurement, samples were kept in an electric oven at 70 °C for 72 h, and then dry weight was measured using a digital scale. For measuring root fresh and dry weights, plants were gently removed from the pots with the roots, and gently washed by a mild stream of water under tap until complete soil removal from the roots. After measuring the fresh weight of the roots, they were transferred to an oven at 70 °C for dry weight measurements [16].

#### Appearance quality (salt stress index: SSI)

During the flowering stage, salt stress symptoms were evaluated based on visual observations of the leaves and flowers with scores ranging from 1 to 5. The scores were determined based on the percentage of damage (Score 1 indicated plants without SSI symptoms, score 2 showed low SSI with damage rate between 1 and 10%, score 3 represented intermediate SSI with damage rate between 11 and 25%, score 4 indicated high SSI with damage rate between 25 and 50% and score 5 showed the highest SSI with damage rate of higher than 50%.). From these scores, the appearance quality index (SSI) was calculated based on established relationships. nSSI = SSI number or SSI score, NSSI = the number of flowers with SSI symptoms and NT = the number of plants with SSI index [16].

1 $$\:\varvec{S}\varvec{S}\varvec{I}\:\varvec{I}\varvec{n}\varvec{d}\varvec{e}\varvec{x}=\sum\:\frac{(nSSI\times\:NSSI)}{NT}$$

#### Leaf relative water content (RWC) and electrolyte leakage content

To assess leaf water status, RWC was assessed. Five leaves from each experimental unit were separated, and the fresh weight (FW) of each leaf was measured. The leaves were then dipped in distilled water for 24 h, and after drying, the saturated or turgid weight of the leaves was measured (TW). Subsequently, the leaves were kept in an oven at 70 °C for 24 h, and their dry weight was recorded (DW). The RWC was calculated according to the formula following Saeed et al. (2011) [17].

2 $$\:\varvec{R}\varvec{W}\varvec{C}\varvec{\%}=\left(\frac{FW-DW}{TW-DW}\right)\times\:100$$

For electrolyte leakage (EC), the middle leaves of plants under stress were punched into ten round pieces with a diameter of one centimeter each. The samples were then placed in test tubes containing 10 mL of distilled water and left at room temperature for 24 h. The amount of EC was measured using the difference in electrical conductivity method following Lutts et al. (1996) [18]. Leaves were excised and washed with deionized water. After drying with filter paper, 1 g fresh weight of leaves were cut into small pieces (about 1 cm^2^) and then immersed in 20 mL deionized water and incubated at 25 °C. After 24 h, electrical conductivity (EC1) of the bathing solution was recorded. These samples were then autoclaved at 120 °C for 20 min to completely kill the tissues and release all electrolytes. Samples were then cooled to 25 °C and the final electrical conductivity (EC2) was measured. The EC was expressed following the formula (Eq. 3):

3 $$\:\varvec{E}\varvec{C}\varvec{\%}=\left(\frac{EC1}{EC2}\right)\times\:100$$

#### Hydrogen peroxide content (H2O2) malondialdehyde content (MDA)

The amount of hydrogen peroxide in plant leaves was measured using the reaction with potassium iodide (KI) [19]. The 0.2 g of leaves were ground with 80% acetone, and centrifuged, and the resulting extract was reacted with a mixture containing potassium phosphate and potassium iodide. Subsequently, the absorption of the samples at 390 nm was measured using a spectrophotometer (SCINCO, S-3100), and the H_2_O_2_ content was calculated based on the standard curve.

To evaluate membrane lipid peroxidation, the MDA content was measured using the method by Due and colleagues (1992) [20]. The powdered leaf tissue was mixed with a mixture of TCA and TBA after liquid nitrogen treatment, and the MDA content was measured. Finally, the absorption of the samples at 532 nm was read using a spectrophotometer (SCINCO, S-3100).

#### Free proline content and total protein

The method by Bates and colleagues (1973) [21] was used to measure free proline in plant leaves. The powdered leaves were mixed with the sulfosalicylic acid solution, and the supernatant underwent a ninhydrin and glacial acetic acid mixture treatment. The reaction was then measured at 520 nm using a spectrophotometer (SCINCO, S-3100), and the absorption levels were plotted against the concentration curve.

For obtaining the extract, 0.5 g of leaf tissue was ground in a 50 mM potassium phosphate buffer (pH = 7). The extracts were separated by a refrigerated centrifuge at 4 °C. The clear extract was used for protein and enzyme quantification. To determine the amount of tissue protein, the protein extract was mixed with a biuret reagent and then the absorption of the samples at 595 nm was measured using a spectrophotometer (SCINCO, S-3100).

#### Chlorophyll and carotenoid content

To assess the total chlorophyll and carotenoid content, 1.0 g of prepared samples from plant leaves was homogenized with 10 mL of 80% acetone to form a uniform solution. The mixture was then centrifuged at 5000 rpm for 5 min. The absorbance of the light-transmitting solution was read using a spectrophotometer at wavelengths of 645 and 663 nm for chlorophyll and wavelengths of 510 and 480 nm for carotenoid. Finally, the concentrations of photosynthetic pigments were calculated in mg g kg^− 1^ using the equations provided [22].

4 $$\:\varvec{T}\varvec{o}\varvec{t}\varvec{a}\varvec{l}\:\varvec{C}\varvec{h}\varvec{l}\varvec{o}\varvec{r}\varvec{o}\varvec{p}\varvec{h}\varvec{y}\varvec{l}\varvec{l}\:=\frac{(20.2\:\left(A645\right)\:+8.02\:\left(A663\right))\times\:V}{(W\times\:100)}$$

5 $$\:\varvec{T}\varvec{o}\varvec{t}\varvec{a}\varvec{l}\:\varvec{C}\varvec{a}\varvec{r}\varvec{o}\varvec{t}\varvec{e}\varvec{n}\varvec{o}\varvec{i}\varvec{d}\:=\frac{(7.60\:\left(A480\right)-1.49\:\left(A510\right))\times\:V}{(W\times\:100)}$$

#### Leaf K^+^and Na^+^content

To measure K^+^and Na^+^ in dried flower leaf samples, 10 fully mature leaves were dried at 105 °C in a hot air oven (ACE400L-60DH) for 48 h, then ground in a mixer and 2 g of plant material was mineralized with a mixture of H_2_SO_4_ and H_2_O_2_ (2:1). Then, the samples were placed in a warm water bath, and the extract obtained after filtration was measured using a flame photometer (JENWAY-PFP7). The K^+^ and Na^+^ concentrations were calculated in mmol kg^− 1^ DW [23, 24].

#### CAT, GPOD, SOD and PAL enzymes activity

The reaction of hydrogen peroxide breakdown with decreased absorption at 240 nm was used to assess CAT enzyme activity. The mixture included potassium phosphate buffer, oxygen water, and enzyme extract, and the CAT enzyme activity was calculated using an extinction coefficient of 39.4 mM^− 1^ cm^− 1^ [25].

The POD enzyme decomposes hydrogen peroxide through the oxidation of substrates like phenolic compounds or antioxidants. Guaiacol is used as an electron donor for enzyme activity measurement. This enzyme, also known as guaiacol peroxidase, was measured at room temperature using a spectrophotometer (SCINCO, S-3100), and the results were reported in units per gram of fresh weight per minute [25].

SOD enzyme activity was measured using the nitro blue tetrazolium (NBT) light reduction inhibition at 560 nm. The samples were prepared with a reaction mixture containing phosphate buffer, NBT, Na-EDTA, riboflavin, methionine, and enzyme extract, exposed to sunlight at 25 °C (w40). After 8 min, the reaction was stopped. The difference in absorption between the samples and the control at 560 nm indicated the inhibition of NBT light reduction in the presence of the SOD enzyme in the sample. By calculating this absorption difference, the enzyme activity was expressed as U g^− 1^ FW [26].

The activity of the PAL enzyme, as a component in the phenolic compounds pathway and enhancement of plant antioxidant system, was measured. In this method, an enzyme extract was combined with an extraction buffer (containing PVP and EDTA) and phenylalanine. The reaction was stopped, and the absorption at 260 nm was measured to convert enzyme activity to enzyme units [27].

#### Gibberellin (GA) and abscisic acid (ABA) content

The extraction process using methanol/water/acetic acid helps to extract a variety of compounds from plant tissues, while freeze-drying preserves compound integrity. Using 80% acetonitrile and 5% formic acid for extraction allows for the extraction of different compounds, and solid-phase extraction cartridges clean up and concentrate the extracts before analysis. The UHPLC-MS/MS method combines high-performance liquid chromatography with mass spectrometry for the separation, identification, and quantification of compounds in complex samples with high sensitivity and specificity [28].

### Statistical analysis

This research was conducted as a factorial experiment with a completely random design with 4 replications. The first factor included different concentrations of PUT treatment (0, 1, 2, and 4 mM) and the second factor included different levels of salt stress with sodium chloride (0, 50, and 100 mM NaCl). Tukey’s test was used to compare the means at a significance level of 5%, and statistical analysis was performed using SPSS software version 19.0. Also, the figures were created by Excel software version 2020. The principal component analysis (PCA) was performed by Origin 2021 software.

## Results and discussion

### Results

#### Effect of treatments on plant height and salt stress index (SSI)

**Fig. 1:**
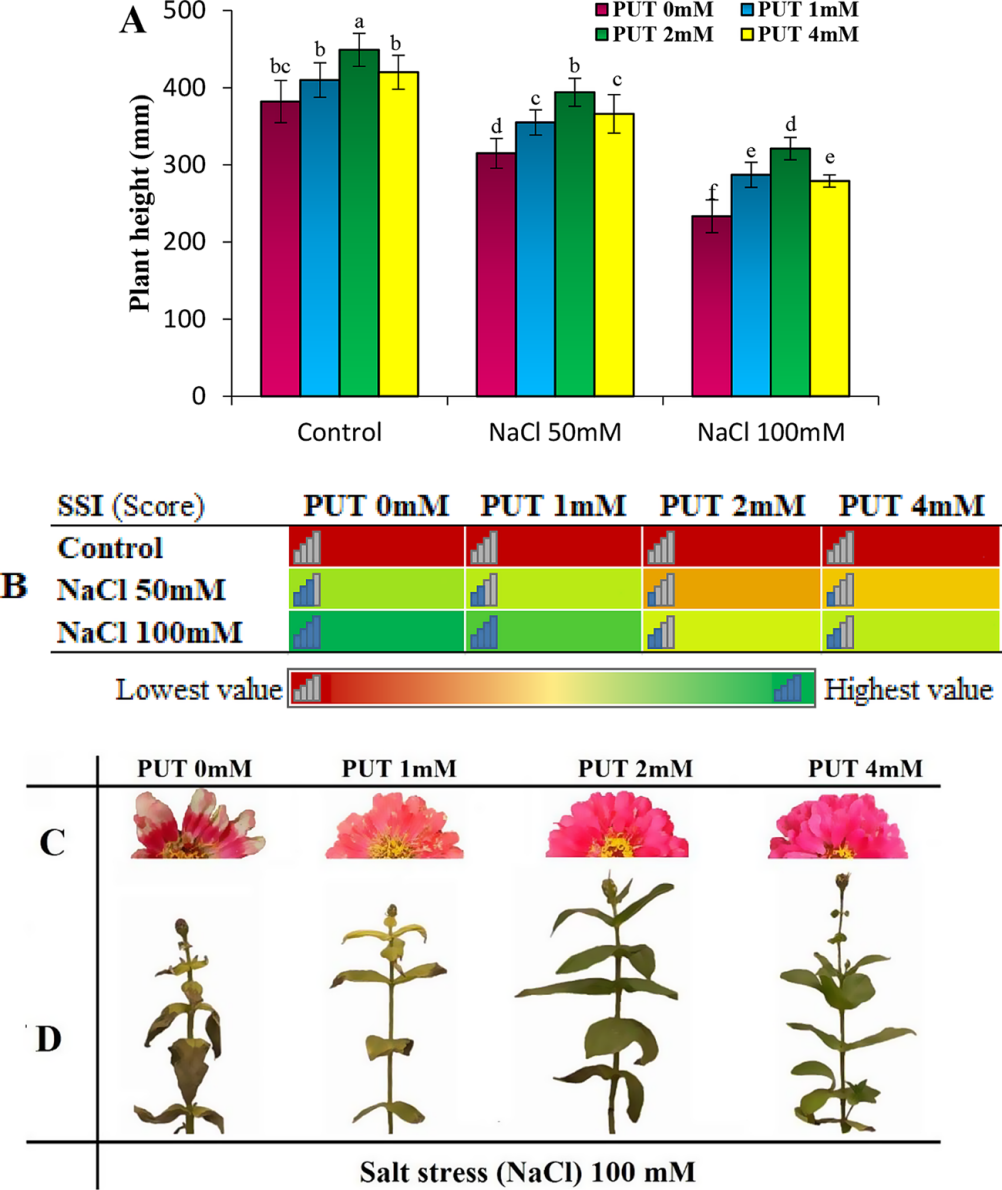
The effect of different levels of putrescine (PUT) treatments on zinnia plant height (**A**) and heat map of SSI (salt stress index; **B**) in Zinnia flowers under two levels of salt stress including 50 and 100 mM. Figure **C** shows the condition of the flowers and **D** shows the condition of the plants under salt stress conditions (NaCl 100 mM). (Means with at least one common letter (in Fig. A) do not have a significant difference at the 5% level in the Tukey test)

The results indicated that under non-salt stress conditions (control), the highest plant height was associated with the 2 mM PUT treatment. There was no significant difference in plant height among different PUT treatments and the untreated control samples (PUT 0 mM). However, with the increasing salt stress, although plant heights decreased, all PUT treatments had taller plants compared to the PUT 0 mM. At salt levels of 50 and 100 mM, the highest plant height was observed with the 2 mM PUT treatment, showing an increase in plant height of over 20% and 27% compared to the PUT 0 Mm at salt levels of 50 and 100 mM, respectively. Subsequently, the 4 and 1 mM PUT treatments had significantly taller plants without a meaningful difference between them compared to PUT 0 mM (Fig. 1A). The results related to the salt stress index also revealed an increase in the index with higher salt stress levels and the occurrence of stress symptoms in plants. The highest value of this index, indicative of the most severe stress symptoms such as leaf scorching, leaf deformation, and reduced plant growth, was observed at the salt stress level of 100 mM under the 0 mM PUT treatment. The results demonstrated that PUT treatments effectively reduced the effects of stress and helped decrease the salt stress index in treated plants. Based on the results, the 2 mM PUT treatment reduced the salt stress index by more than 28% and 35% at salt levels of 50 and 100 mM, respectively, preserving the visual quality of the flowers under salt stress conditions. However, the statistical analysis did not show a significant difference between this treatment and the 4 mM PUT treatment (Fig. 1B). Figure 1C and D shows the appearance of flowers and plants under salt stress (NaCl 100 mM). In these figures, the treatment of PUT 0 mM has the lowest quality and the treatments of PUT 2mM and PUT 4mM have the highest appearance quality (Fig. 1C and D).

#### Effect of treatments on plant biomass traits

Mean comparisons showed that plant biomass traits were significantly affected by increasing the level of salt stress, as with the increase in salt stress level, the weight of fresh and dry shoots, number and surface area of leaves, flower diameter, and length of internodes decreased. However, PUT treatments were able to improve these traits compared to PUT 0 mM. In most of these traits, the highest values without a significant difference among them were related to the PUT treatments of 2 and 4 mM, while the lowest was observed in PUT 0 mM, although a statistically significant difference was not observed between PUT 0 mM and PUT 1 mM treatment (Table 1).

**Table 1:** Effect of putrescine treatments on some morphological indices of Zinnia flowers under salt stress

Salt stress (mM)	PUT (mM)	Plant FW (g)	Plant DW (g)	Leaf numbers	Leaf area (mm^2^)	Flower diameter (mm)	Internode length (mm)
**Control (0)**	**0**	25.28^ab*^	3.55^ab^	12.66^bc^	632.20^a^	46.26^a^	26.03^a^
	**1**	25.73^ab^	3.57^ab^	14.00^ab^	638.33^a^	46.43^a^	26.10^a^
	**2**	26.02^a^	3.67^a^	15.33^a^	651.01^a^	47.03^a^	27.11^a^
	**4**	25.96^a^	3.66^a^	15.33^a^	651.11^a^	47.32^a^	26.96^a^
**NaCl 50**	**0**	23.15^cd^	3.32^cd^	9.33^de^	622.34^a^	37.03^c^	20.62^c^
	**1**	23.63^bc^	3.33^bc^	10.66^cd^	625.66^a^	38.20^bc^	20.73^bc^
	**2**	24.73^ab^	3.45^abc^	12.00^bc^	636.47^a^	40.16^b^	22.56^b^
	**4**	24.66^abc^	3.47^abc^	12.66^bc^	637.25^a^	39.98^b^	22.74^b^
**NaCl 100**	**0**	17.45^f^	2.69^f^	6.66^f^	491.74^c^	30.33^d^	14.45^e^
	**1**	18.36^ef^	2.80^ef^	7.33^ef^	505.81^c^	31.46^d^	17.16^d^
	**2**	20.16^de^	3.00^de^	9.33^de^	549.92^b^	35.33^c^	18.73^cd^
	**4**	19.91^e^	3.07^de^	9.33^de^	548.66^b^	35.16^c^	19.36^c^

*In every column, means with at least one common letter do not have a significant difference at the 5% level in the Tukey test

#### Effect of treatments on physiological and biochemical traits

**Fig. 2:**
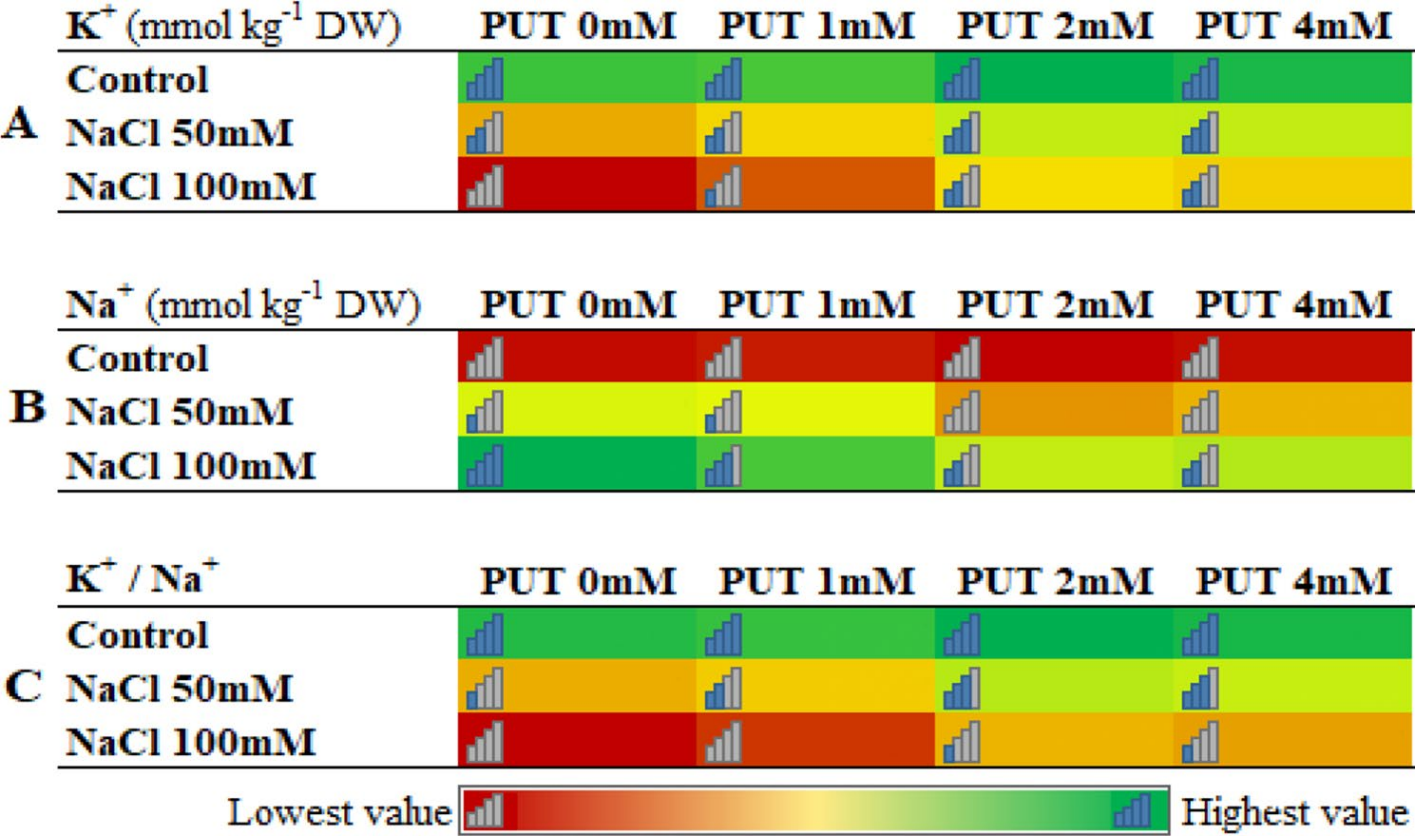
Heat map of effect of putrescine treatments on the K^+^ (potassium; **A**), Na^+^ (sodium; **B**), and K^+^/Na^+^ content of Zinnia leaves under different levels of salt stress

The results related to the RWC showed that although this index decreased in flowers with an increase in salt stress level, PUT treatments were able to effectively maintain the RWC in the leaves of Mallow plants. Among them, the 4 mM PUT treatment had a better effect compared to other treatments, although the difference with the 2 mM PUT treatment was not statistically significant (Table 1). Additionally, with an increase in salt stress level up to 100 mM, EC, H_2_O_2_ content, MDA, and proline content increased, and the protein, chlorophyll, and carotenoid content decreased. However, PUT treatments effectively protected plants against salt stress, with lower levels of electrolyte leakage, hydrogen peroxide, malondialdehyde, and proline compared to the PUT 0 mM, and higher content of protein, total chlorophyll, and carotenoids. The results showed that in most of the studied traits, the 2 and 4 mM PUT treatments significantly improved these traits compared to the 1 mM and 0 mM treatments and had a greater effect in reducing the effects and symptoms of salt stress (Table 2). Investigation of leaf K^+^ and Na^+^ content also showed that with an increase in salt stress level, K^+^ content decreased and Na^+^ content increased, but PUT treatments had higher K^+^ content and K^+^/Na^+^ ratio in all salt levels compared to untreated plants (0 mM PUT). The highest amount of K^+^ content and K^+^/Na^+^ ratio and lowest Na^+^ was observed in the 2 and 4 mM treatments of PUT, followed by 1 mM treatment of PUT, in which PUT 0 mM had no significant difference with PUT 0 mM (Fig. 2).

**Table 2:** Effect of putrescine treatments on some physiological indices of Zinnia flowers under salt stress

Salt stress (mM)	PUT (mM)	RWC (%)	EC (%)	H_2_O_2_ (mmol^− 1^ kg FW)	MDA (µmol^− 1^ kg FW)	Proline (mmol^− 1^ kg FW)	Protein (g kg^− 1^ FW)	Total chlorophyll (g kg^− 1^ FW)	Carotenoid (g kg^− 1^ FW)
**Control (0)**	**0**	85.26^ab*^	20.69^f^	1.69^fg^	1.72^f^	0.77^fg^	1.90^ab^	0.26^a^	0.072^a^
	**1**	85.43^ab^	20.53^f^	1.50^gh^	1.65^f^	0.76^fg^	1.91^ab^	0.27^a^	0.073^a^
	**2**	87.72^a^	19.46^f^	1.28^h^	1.61^f^	0.68^g^	2.03^a^	0.28^a^	0.074^a^
	**4**	87.79^a^	19.15^f^	1.31^h^	1.62^f^	0.70^fg^	2.01^a^	0.28^a^	0.074^a^
**NaCl 50**	**0**	75.21^de^	36.77^c^	2.23^d^	3.26^cd^	1.10^cd^	1.55^cd^	0.15^c^	0.059^cd^
	**1**	77.26^cd^	33.64^d^	2.05^de^	2.96^de^	1.02^de^	1.63^c^	0.16^c^	0.061^bc^
	**2**	80.01^bc^	29.70^e^	1.85^ef^	2.62^e^	0.88^ef^	1.86^b^	0.20^b^	0.069^ab^
	**4**	79.90^bc^	29.52^e^	1.91^ef^	2.64^e^	0.87^efg^	1.87^b^	0.21^b^	0.070^ab^
**NaCl 100**	**0**	66.74^f^	46.61^a^	4.52^a^	4.77^a^	1.91^a^	1.18^f^	0.10^d^	0.040^f^
	**1**	67.41^f^	41.16^b^	4.18^b^	4.23^b^	1.70^b^	1.33^e^	0.11^d^	0.044^ef^
	**2**	69.86^e^	35.63^cd^	3.68^c^	3.65^c^	1.29^c^	1.47^d^	0.17^c^	0.052^de^
	**4**	70.22^e^	36.84^c^	3.55^c^	3.69^c^	1.28^c^	1.48^d^	0.16^c^	0.051^de^

* In every column, means with at least one common letter do not have a significant difference at the 5% level in the Tukey test

#### Effect of treatments on enzyme activity

**Fig. 3:**
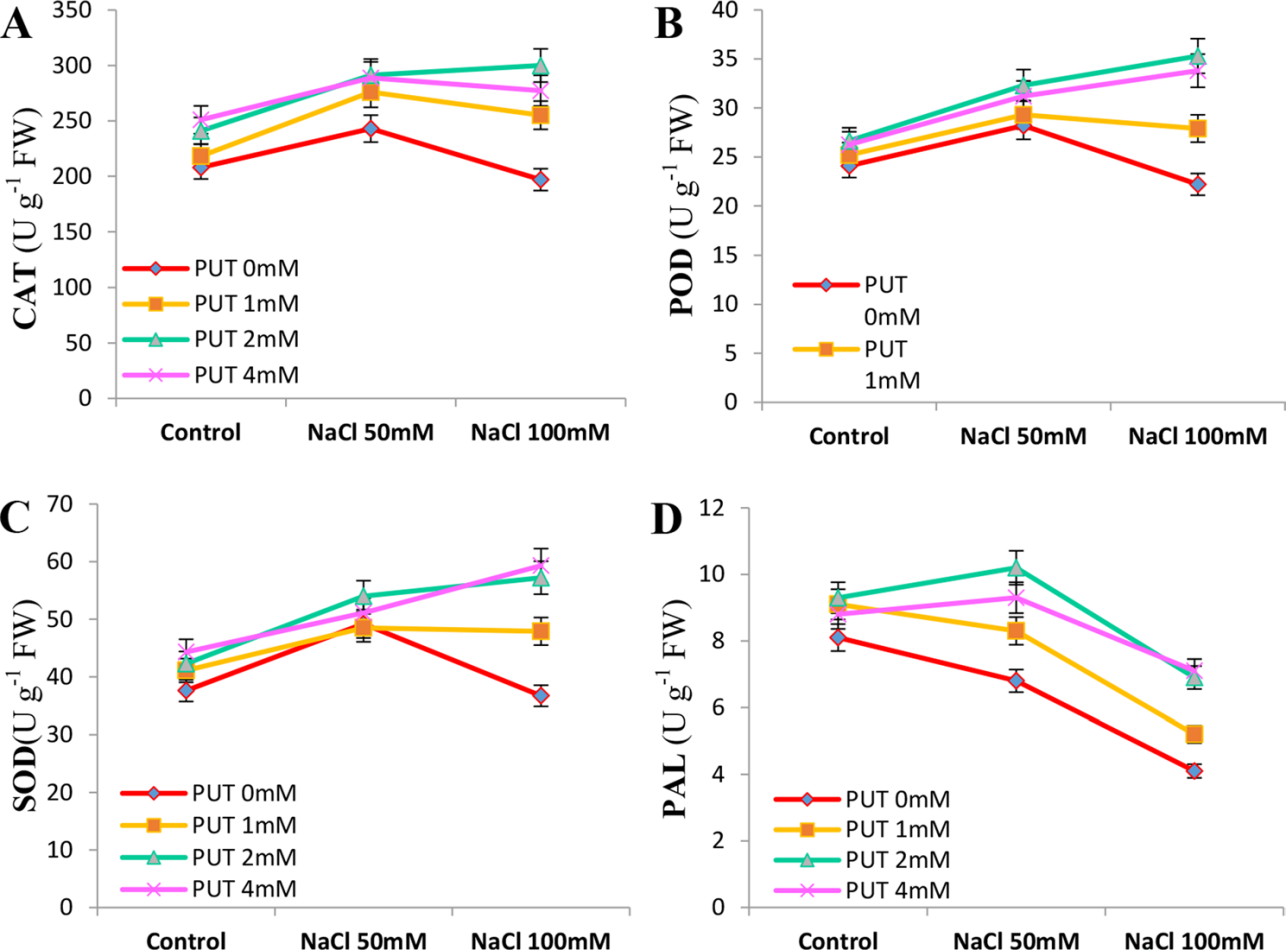
Effect of putrescine treatments on the activity of catalase (CAT; **A**), peroxidase (POD; **B**), superoxide dismutase (SOD; **C**), and phenylalanine ammonia-lyase (PAL; **D**) enzymes in Zinnia flowers under two different levels of salt stress (Means with at least one common letter do not have a significant difference at the 5% level in the Tukey test)

In this study, to investigate antioxidant activity in flowers under salt stress, the activity of some antioxidant enzymes was studied. The results showed that the CAT enzyme activity increased in the 50 mM salt stress level in treatments compared to the non-stress conditions (control), then decreased at the 100 mM stress level. However, PUT treatments of 1, 2, and 4 mM had higher CAT activity compared to the PUT 0 mM samples, with the highest activity of this enzyme observed in the 2 mM PUT treatment. The results of the GPOD enzyme showed that the activity of this enzyme initially increased in the 50 mM salt stress level in the PUT 0 mM and 1 mM samples, then decreased at the 100 mM salt stress level, but in other PUT treatments, the activity of this enzyme increased with increasing salt stress level, with the highest activity observed in the 2 and 4 mM PUT treatments (Fig. 3. A, B).

The results related to the SOD enzyme were similar to the GPOD enzyme, as with an increase in salt stress level, the activity of this enzyme decreased in PUT 0 mM, but increased in the PUT 1, 2, and 4 mM treatments. In the 100 mM salt stress level, the 2 and 4 mM treatments had the highest activity, while PUT 0 mM had the lowest. The results of the PAL enzyme also showed that with an increase in salt stress level, the activity of this enzyme initially increased in the 2 and 4 mM PUT treatments at the 50 mM salt stress level, then decreased at the 100 mM salt stress level. Meanwhile, the activity of this enzyme in the 1 mM PUT and PUT 0 mM treatments decreased with increasing salt stress level, with the lowest activity observed in these treatments at the 100 mM salt stress level (Fig. 3.C, D).

#### Effect of treatments on ABA and GA content

**Fig. 4:**
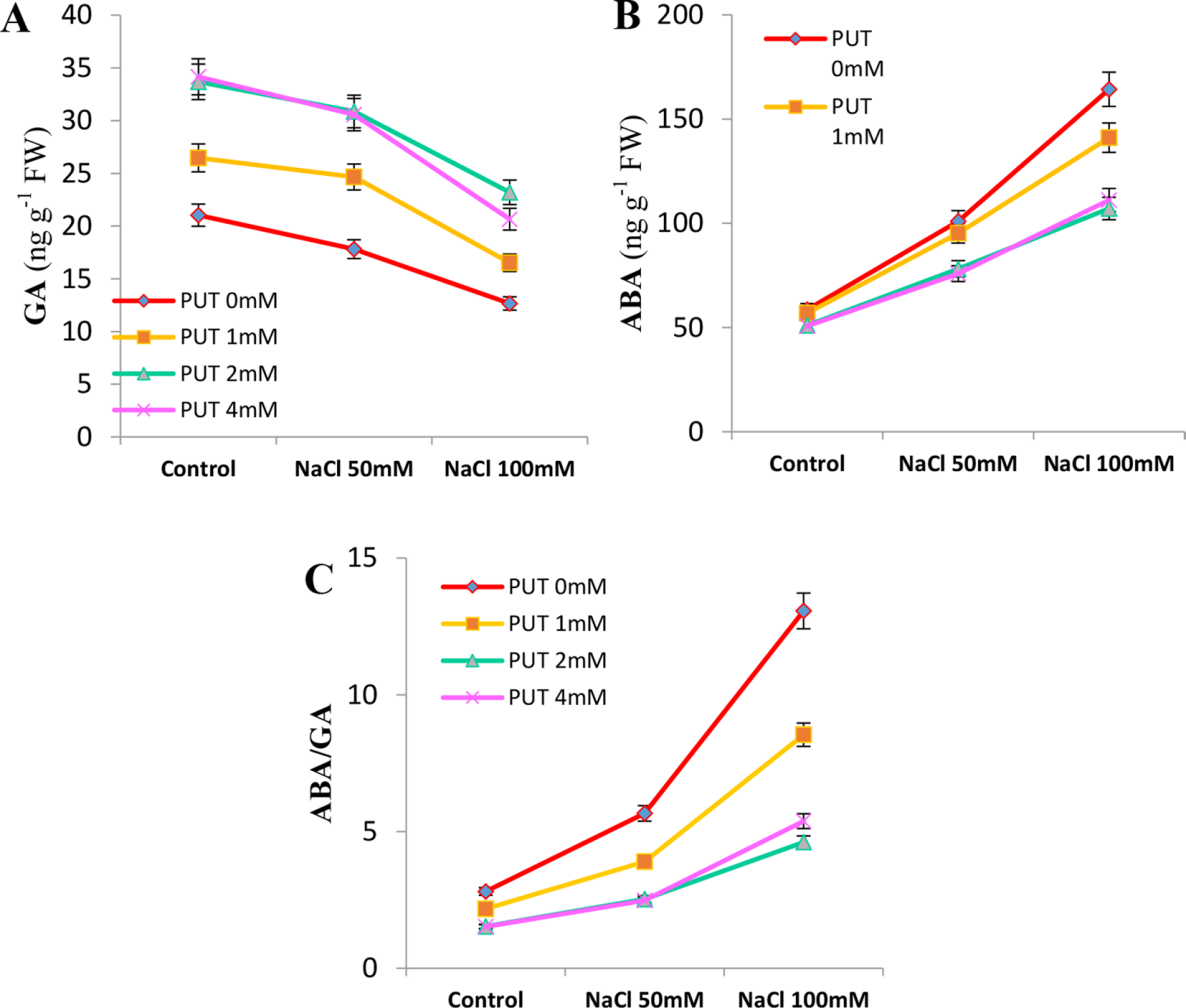
Effect of putrescine treatments on gibberellin content (GA; **A**), abscisic acid content (ABA; **B**), and the ratio of abscisic acid to gibberellin (**C**) in Zinnia flowers under two different levels of salt stress (Means with at least one common letter do not have a significant difference at the 5% level in the Tukey test)

In this experiment, the content of ABA and GA, as the most important PGRs inhibiting growth and stimulating growth, respectively, were studied. Since the ratio of these two PGRs is also crucial in determining the final growth of the plant, we also investigated their ratio. The results showed that with an increase in salt stress level, the ABA content increased and the GA content decreased, leading to an increase in the ABA/GA ratio in Mallow plants under salt stress. In this study, PUT treatments effectively prevented the increase in ABA and decrease in GA, with the lowest ABA content and highest GA content observed in the 2 and 4 mM PUT treatments, and no significant difference observed between these treatments. This resulted in the plants treated with 2 and 4 mM PUT having a lower ratio of ABA/GA compared to the 1 mM PUT treatment and PUT 0 mM. Additionally, in the next order, the samples treated with 1 mM PUT had lower ABA, higher GA, and a lower ratio of ABA/GA compared to the plants treated with PUT 0 mM at different salt levels (Fig. 4.A, B, C).

#### PCA for morphophysiological and biochemical characteristics

**Fig. 5:**
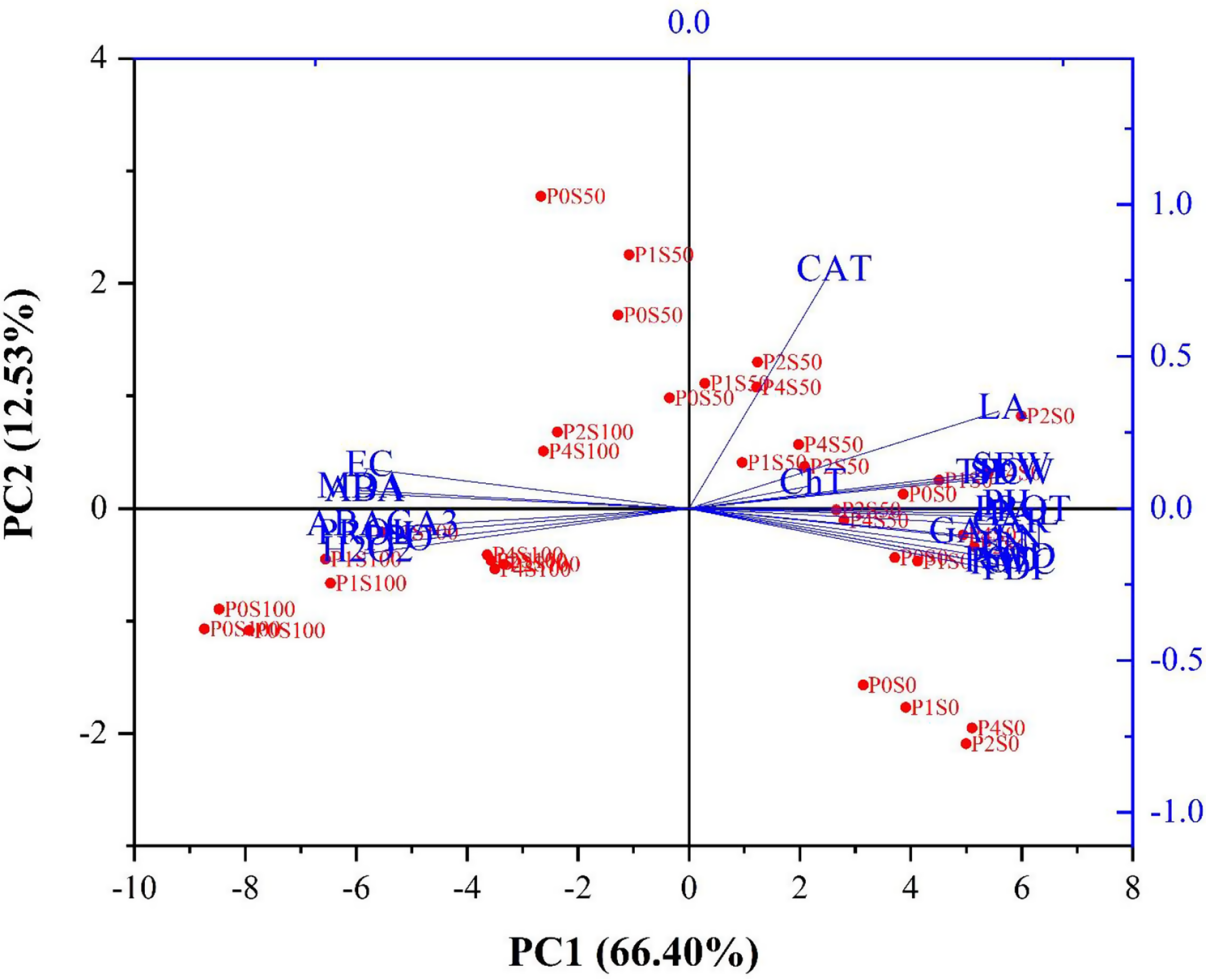
PCA biplot of the analyzed parameters in Zinnia after exposure to PUT under salinity conditions. *Plant height (PH), Shoot fresh weight (SFW), Shoot dry weight (SDW), Leaf number (LN), Leaf area (LA), Flower diameter (FDI), Internode length (EN), Relative Water Content (RWC), Proline (Prol), Protein (Prot), Total chlorophyll (ChT), Carotenoid (CAR), Malondialdehyde (MDA), catalase (CAT), peroxidase (POD), superoxide dismutase (SOD), phenylalanine ammonium lyase (PAL), Total phenol (TFC), Ratio of potassium to sodium (K+/Na+), catalase (CAT), peroxidase (POD), superoxide dismutase (SOD), phenylalanine ammonium lyase (PAL), Total phenol (TFC), Hydrogen Peroxide Content (H2O2), Gibberellin (GA) and Abscisic Acid (ABA) content

In this research, Table 3; Fig. 5 illustrates the principal component analysis (PCA) of the studied characteristics of Zinnia. Different treatments with PUT and salt stress, as well as various traits under study, are grouped accordingly. The first three main components main components explain 91.04% of the total variance, with the first principal component representing 66.40% and the second principal component representing 12.53% of the total variance. The loading plot results demonstrate that characteristics such as POD, SOD, ABA and RWC exhibit strong loadings in the first and second components. These characteristics can be seen as representative of the effects of different concentrations of PUT under salt stress on the morphophysiological and biochemical characteristics of Zinnia (Fig. 5).

**Table 3:** Eigenvalues of the principal component axes from the multiple regression analysis of the morphophysiological and biochemical characteristics in Zinnia under the influence of different concentrations of PUT under salinity conditions

Traits	Component		
	1	2	3
*PH	0.848	0.314	0.206
SFW	0.786	0.415	0.335
SDW	0.806	0.405	0.291
LN	0.844	0.114	0.403
LA	0.686	0.604	0.349
FDI	0.951	0.106	0.178
EN	0.892	0.178	0.331
RWC	0.942	0.124	0.186
EC	-0.913	-0.170	-0.280
Prol	-0.836	-0.402	-0.302
Prot	0.847	0.258	0.417
ChT	0.086	-0.011	0.893
CAR	0.876	0.252	0.232
MDA	-0.896	-0.241	-0.330
CAT	0.123	0.972	-0.003
POD	0.951	0.160	0.020
SOD	0.959	0.157	0.154
PAL	0.885	0.276	0.306
TFC	0.671	0.313	0.556
K^+^/Na^+^	-0.666	-0.317	-0.568
H_2_O_2_	-0.812	-0.460	-0.296
GA	0.781	0.189	0.141
ABA	-0.925	-0.287	-0.176
ABA/GA	-0.844	-0.396	-0.124
Eigenvalues	15.93	3.01	2.91
% of variance	66.40	12.53	12.11
Cumulative %	66.41	78.93	91.04

*Plant height (PH), Shoot fresh weight (SFW), Shoot dry weight (SDW), Leaf number (LN), Leaf area (LA), Flower diameter (FDI), Internode length (EN), Relative Water Content (RWC), Proline (Prol), Protein (Prot), Total chlorophyll (ChT), Carotenoid (CAR), Malondialdehyde (MDA), catalase (CAT), peroxidase (POD), superoxide dismutase (SOD), phenylalanine ammonium lyase (PAL), Total phenol (TFC), Ratio of potassium to sodium (K^+^/Na^+^), catalase (CAT), peroxidase (POD), superoxide dismutase (SOD), phenylalanine ammonium lyase (PAL), Total phenol (TFC), Hydrogen Peroxide Content (H_2_O_2_), Gibberellin (GA) and Abscisic Acid (ABA) content

## Discussion

**Fig. 6:**
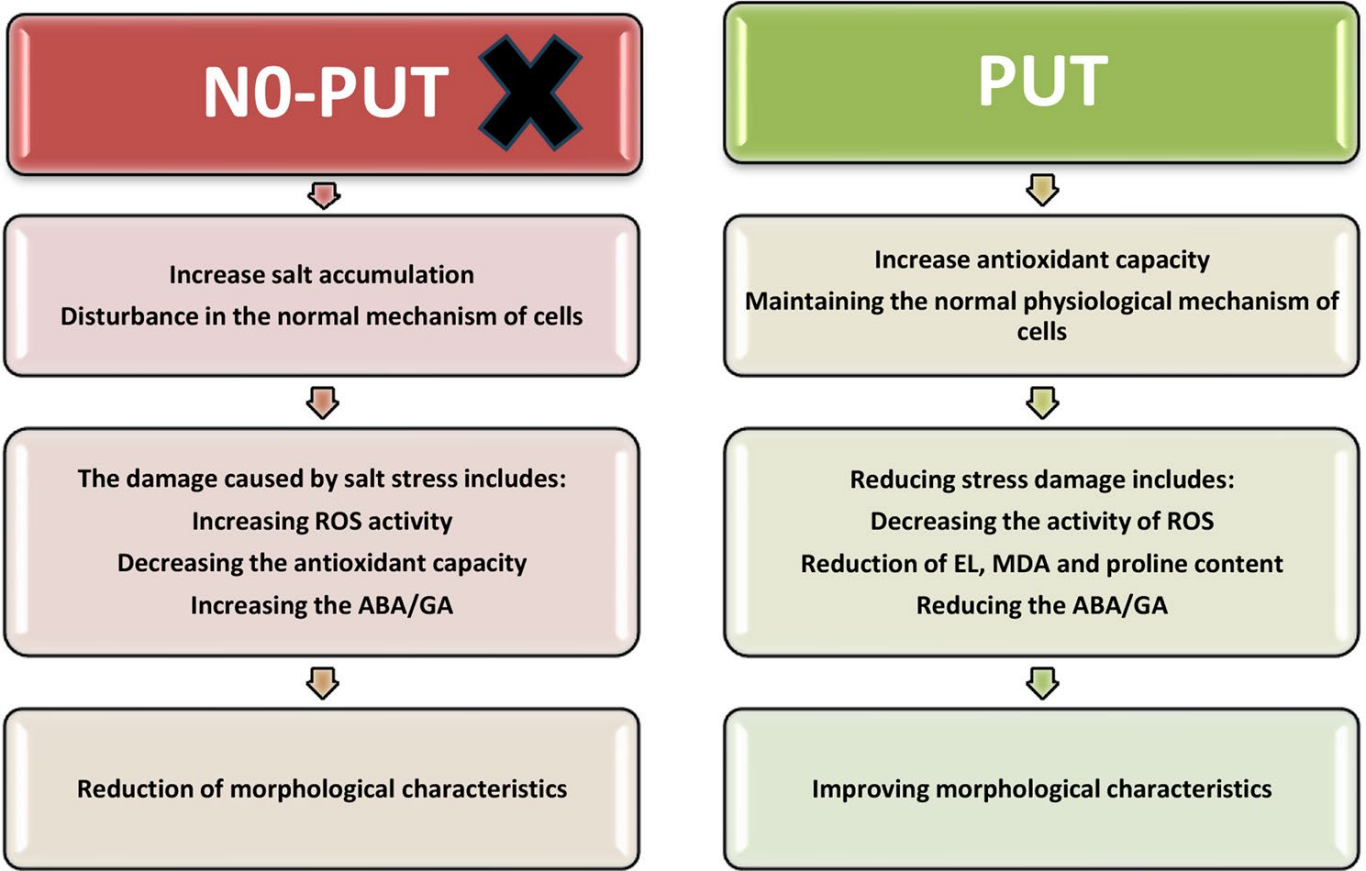
Proposed schematic mechanism of the effect of PUT treatment on Zinnia plants under salt stress. This figure illustrates that under conditions where PUT is absent, salt accumulation disrupts the normal functioning of plants, leading to salt stress damage and a decrease in the morphological quality of Zinnia flowers. However, the application of PUT treatment enhances the antioxidant system and reduces ROS activity, thus preserving the normal functioning of cells under salt stress conditions and improving plant morphological indicators under salt stress compared to the time when PUT was not present (control)

This research investigates the effect of PUT on increasing the resistance of plants to salt stress and its impact on quantitative and qualitative characteristics. The results indicated a decrease in quantitative and qualitative characteristics of plants under high levels of stress; however, the use of PUT reduces the negative effects of salt stress and effectively maintains the quality of Zinnia flowers compared to untreated control samples. Salt stress stress reduces the water potential around the roots, increases the toxicity of ions, especially sodium and chlorine ions, disrupts ion balance in plant cells and tissues, limits water uptake by plants, disrupts the physiological processes of plants, and reduces photosynthetic pigments ultimately reduces growth. On the other hand, the plant has different mechanisms to cope with the undesirable effects of salt stress, including non-absorption or very low absorption of salt by the plant, high tissue and cellular resistance of plants, salt accumulation in vacuoles of cells, separation of absorbed ions, and increase in the surface of some substances and biochemical compounds in cells [29]. Figure 6 shows the suggested schematic of the effect of PUT treatments in the present study. In this figure, the mechanisms of PUT treatment in reducing salt stress damage and maintaining the quality of zinnia flowers have been compared with untreated control samples (Fig. 6).

To mitigate the negative effects of salt stress, the findings of this study suggest that treatments with polyamines (PUT) may counteract growth inhibition by enhancing antioxidant enzyme activity and reducing the ABA/GA ratio. This resulted in improved growth of treated plants compared to untreated controls, as evidenced by higher K^+^ content and K^+^/Na^+^ ratio, increased height, enhanced morphological characteristics, reduced EL and salt stress index in Zinnia plants exposed to salt stress conditions. Research has shown that sodium ion accumulation in plant tissues can lead to a significant reduction in K^+^ content, resulting in leaf necrosis and reduced photosynthesis rates [30]. Also add the evidence where PUT improves the quality of the ornamental plants under stress conditions A study examined the impact of putrescine on mycorrhizal colonization, growth, and flowering in Gerbera in a greenhouse. Findings revealed enhanced mycorrhizal colonization with 2 mM putrescine, leading to improved flower quality and reduced oxidative stress effects [15, 31].

A reduction in K^+^ content in plants under salt stress may be due to non-absorption of water due to sodium accumulation inside the cells, but in PUT treatments with reduced detrimental effects of salt stress, plants had higher water absorption and higher K^+^ content and K^+^/Na^+^ ratio compared to untreated plants. The results related to RWC also confirm the claim that PUT treatments had higher RWC compared to untreated plants. Therefore, one of the main reasons for the significant reduction in salt stress in treated plants in the present study may be due to higher K^+^ content [32]. Research indicates that the application of salt stress on seedlings causes a noticeable reduction in their growth and development, which is recognized as one of the main obstacles in the horticultural sector. The negative effects of salt stress stress, observed as a reduction in the size and weight of seedlings, can significantly affect morphological, and physiological characteristics as well as marketable product indicators (freshness and visual quality) during the plant growth period [13, 33].

In this regard, the application of PUT as a polyamine can improve the ability of plants to withstand salt stress stress due to its multiple roles. This substance, by helping to maintain osmotic balance inside the cells, strengthening cellular membrane stability, reducing oxidative stress by increasing antioxidants, regulating stress-related PGRs, and supporting photosynthetic activities, allows plants to better adapt to saline and unfavorable environmental conditions. It can also help increase the plant’s ability to absorb and effectively use water and nutrients such as potassium and calcium by stabilizing the cellular membrane, which helps maintain cell health and reduce damage from oxidative stress, as a result, PUT helps to increase the height and improve the weight of fresh and dry plants under salt stress stress conditions [13]. Under stressful conditions, hormonal imbalance and increased ABA lead to sodium ion accumulation in plant cells, causing stress in plants. This process is usually accompanied by the production of active forms of oxygen, especially H_2_O_2_, which is considered a common indicator of stress [34]. The use of PUT leads to a reduction in the ABA/GA ratio, resulting in treated plants exhibiting greater growth and improved morphological traits. Numerous studies have reported that PUT moderates the hormonal balance of plants [35]. ABA is a PGR that typically increases with environmental stressors, generally exacerbating stress, whereas GAs are associated with growth and development. Balancing these two PGRs can potentially enhance stress resistance and improve the growth and development of plant cells. PUT use increases the activity of antioxidants, reducing the damaging effects of free radicals [33, 35, 36]. Additionally, this polyamine plays a role in reducing the ratio of ABA/GA, leading to a better hormonal signaling balance, which can promote better plant adaptability to combat environmental stresses [36].

The stress caused by hormonal imbalance can lead to reduced water permeability from the roots, decreased water density in leaves, and increased RWC in plants. Increased RWC in these conditions indicates the plant’s ability to cope with the adverse effects of salt stress and maintain water balance under challenging conditions. Research results demonstrate that PUT-treated plants show an increase in this component compared to untreated samples. Indeed, PUT treatments, by strengthening the antioxidant system and hormonal balance, lead to a reduction in ABA [37]. Furthermore, the damage resulting from stress and the increase in harmful electrolyte leakage into plant cells under salt stress conditions are significantly mitigated by the application of PUT. In other words, PUT improves cell membrane strength by regulating ions such as potassium and water absorption inside and outside the cell, creating a more stable environment for plant growth. This not only reduces electrolyte leakage but also improves the morphological characteristics of plants. This effectiveness, especially in preventing chloride and sodium leakage into the cell, not only aids in protecting vital plant processes but also enhances plant tolerance to saline conditions [1, 35]. Therefore, electrolyte leakage, especially sodium electrolyte leakage, can lead to damage to cell membranes. Plant cell membranes have an incredibly complex and sensitive structure that is essential for maintaining internal and external cell balance. Electrolyte leakage occurs due to disruptions in the activity of ion pumps and membrane channels, which are normally regulated by hormonal settings and internal cell signals [38]. With the destruction of cell membranes, sodium ions can partially enter the cell. Moreover, increased sodium ion permeability into the cell can stimulate the activity of specific enzymes present in the cell. These activities may lead to the production of H_2_O_2_ and other free radicals, causing oxidative damage to the cell. Consequently, H_2_O_2_ production resulting from increased sodium ion permeability can lead to cell membrane damage and improper cell function. Therefore, the effects of H_2_O_2_ as a fundamental component of salt stress processes in plants have been studied [39]. One of the factors examined regarding H_2_O_2_ is MDA, which is known as a precursor of lipid oxidation thus the increase in MDA as an indicator of one of the prominent responses of plants to salt stress due to increased oxidative stress and ROS production [40].

In the current study, the content of MDA increased with salt stress, indicating a decrease in salt stress resistance and cell membrane damage. However, in PUT-treated plants, cell membranes are protected against lipid peroxidation, and the accumulation of MDA is prevented, which is consistent with the experimental results in 2022, as reported. They stated that PUT plays an important role in regulating the growth and development of plants. This polyamine, with its signaling role, can facilitate the improvement of cell membranes through the regulation of the activities of various enzymes and proteins. Among the mechanisms by which PUT can improve cell membranes are the regulation of ion balance inside and outside the cell, increased activity of antioxidants such as carotenoids, and regulation of the production and activity of enzymes related to cellular structure. These functions extensively enhance the strength and health of cell membranes, leading to better cellular function and consequently improved plant growth. The application of polyamines by enhancing PUT metabolism inhibits MDA accumulation by improving antioxidant activity [41].

In line with reducing H_2_O_2_ accumulation, biochemical evaluations of plants have shown that plant growth under stress conditions leads to the accumulation of low molecular weight compounds called “compatible solutes” in their tissues, which play various and important roles in creating stress resistance. One of the most important compatible solutes in plants is proline [42]. Proline, as a low molecular weight compound, plays an important role in environmental stress adaptation processes. Proline accumulates inside the plant as an amino acid under stress conditions after the breakdown of proteins and the release of amino acids. In the current study, a decrease in proline was observed in PUT-treated plants, which could be due to the enhancement of the plants’ antioxidant system and the reduction of protein breakdown. Also, in untreated plants, besides increasing protein degradation, glutamate can compete for glutamate as a precursor for proline synthesis, leading to an increase in proline and a decrease in chlorophyll due to this competition. Moreover, increased proline in untreated plants, with its osmotic role, can increase osmotic pressure inside the cells, which may inhibit water absorption for chlorophyll synthesis [38, 43]. In the current study, the application of putrescine resulted in a decrease in proline and the development of resistance to salt stress, and the chlorophyll content in treated plants improved. On the other hand, soil salt stress and subsequent osmotic and oxidative stress in plants can lead to protein degradation, which may result in a decrease in protein content in treated plants. The protective role of PUT in cell membranes helps preserve the structure and function of important proteins. Additionally, PUT can act as an inducer of plant defense responses against salt stress. Under stress conditions, these defense responses may include increased synthesis of specific proteins such as heat shock proteins (HSPs) and antioxidant defense enzymes, which were observed in the results of the current study, and PUT treatments had higher protein content [44].

Free radical scavenging by carotenoids, which are important antioxidants in plants, is carried out under salt-stress conditions. The production of free radicals increases under salt stress conditions, and carotenoids help counteract these harmful factors and decrease. PUT may enhance antioxidant responses and thus increase the activity of the antioxidant defense system. This may include an increase in carotenoids. Carotenoids help strengthen biological membranes and may allow cells to exhibit greater resistance to electrolyte leakage caused by salt stress. Plants also attempt to maintain their water balance by reducing the adverse effects of increased salt concentration in the root environment [45]. Enzymes such as CAT, POD, and SOD play key roles in scavenging free radicals and preventing damage to vital molecules such as DNA, proteins, and lipids. In this study, it was observed that under moderate salt stress conditions (50 mM), CAT activity increased, which can be perceived as a protective response against milder oxidative stress. However, at higher salt levels (100 mM), the activity of this enzyme decreased, which may be due to exceeding the plant’s defense system capacity or enzyme degradation due to the intensity of stress [46]. Interestingly, treatment with PUT appears to have increased CAT activity at both salt levels, indicating that PUT may help maintain or strengthen enzyme activities under stress conditions or even stimulate enzymatic activities. The protective role of PUT may result from reducing the production of ROS and reducing the consumption of antioxidants to neutralize them [15]. Similarly, the activities of GPOD and SOD changed differently under high and low concentrations of PUT, indicating the potential protective effects of PUT [47]. Additionally, the activity of an important enzyme, PAL, as a key factor in the biosynthesis of phenolic compounds, has been investigated [15, 38].

PAL plays a crucial role in the initial stage of phenolic compound production from the amino acid phenylalanine. As a result, treatment of plants with PUT and increased activity of this enzyme can lead to increased production and oxidation of phenolic compounds, which may be a reason for the higher phenol content in treated plants and reduced stress damage [15, 48]. Consistent with our results, studies have shown that the use of polyamines, including PUT, enhances stress resistance pathways and reduces polyamine degradation in plants. They concluded that the use of PUT improves the physiological traits of anthurium flowers under cold stress [49]. Additionally, in another experiment, the use of PUT under saline conditions on ginger plants was investigated. They reported that salt stress had detrimental effects on this plant, and the application of PUT led to the accumulation of various protective compounds, including proline, soluble carbohydrates, soluble sugars, and soluble proteins [50].

## Conclusion

The present results showed that with an increase in the level of salt stress, the extent of stress damage increased, and the stress index significantly increased in ornamental Zinnia. However, PUT treatments resulted in a reduction in the salt stress index and maintained the plant quality of Zinnia compared to the untreated plants. Examination of the studied indicators showed that PUT treatments increased K^+^ content, K^+^/Na^+^ ratio, and activity of antioxidant enzymes and reduced the ABA/GA ratio in Zinnia plants under salt stress conditions, leading to a decrease in the salt stress index (SSI), preservation of the visual quality of plants, and an increase in height and morphological traits of Zinnia. Therefore, based on the present results, we recommend the use of 2 mM PUT treatment as a promising practical approach to reduce salt stress damage in Zinnia flowers.

## Data Availability

No datasets were generated or analysed during the current study.

## Abbreviations

PUT: Putrescine (C_4_H_12_N_2_)
K+: Potassium content
Na+: Sodium content
CAT: Catalase
GPOD: Guaiacol peroxidase
SOD: Superoxide dismutase
PAL: Phenylalanine ammonium lyase
ABA: Abscisic acid
GA: Gibberellin
RWC: Leaf relative water content
FW: Fresh weight
DW: Dry weight
EL: Electrolyte leakage
H2O2: Hydrogen Peroxide Content
MDA: Malondialdehyde
SSI: Salt stress index
